# Modeling, the Optimization of the Composition of Emulgels with Ciclopirox Olamine, and Quality Assessment

**DOI:** 10.3390/polym16131816

**Published:** 2024-06-26

**Authors:** Agne Mazurkeviciute, Inga Matulyte, Marija Ivaskiene, Modestas Zilius

**Affiliations:** 1 Institute of Pharmaceutical Technologies, Lithuanian University of Health Sciences, 50162 Kaunas, Lithuania; agne.mazurkeviciute@lsmu.lt (A.M.); inga.matulyte@lsmu.lt (I.M.); 2 Department of Clinical Pharmacy, Lithuanian University of Health Sciences, 50162 Kaunas, Lithuania; 3 Department of Drug Technology and Social Pharmacy, Lithuanian University of Health Sciences, 50162 Kaunas, Lithuania; 4 Dr. L. Kraučeliūnas Small Animal Clinic, Veterinary Academy, Lithuanian University of Health Sciences, 47181 Kaunas, Lithuania; marija.ivaskiene@lsmu.lt

**Keywords:** emulgels, design of experiments, ciclopirox olamine, poloxamer 407, mineral oil

## Abstract

The design and development of pharmaceutical products require specific knowledge, time, and investment. Response surface methodology (RSM) is a widely used technique in the design of experiments (DoE) to optimize various processes and products. The aim of this study was to model and produce experimental emulgels containing 1% ciclopirox olamine and to evaluate their physical, rheological, and mechanical properties and their ability to release ciclopirox olamine. The objective was to optimize the composition of the experimental emulgel containing 1% ciclopirox olamine by applying a central composite design based on selected criteria. The surfactant (polysorbate 80) had the greatest influence on the physical, rheological, and mechanical properties of the emulgels, as well as on the release of ciclopirox olamine from these systems. During the optimization process, an emulgel of optimal composition was generated containing 38.27% mineral oil, 6.56% polysorbate 80, and 55.17% hydrogel containing 1% ciclopirox olamine, meeting specified criteria (dependent variables) including the maximum flux of ciclopirox olamine, the minimum sol–gel transition temperature (T_sol/gel_), and the minimum particle size of the oil phase. The oil phase particle size (D50) of this emulgel was determined to be 0.337 µm, the system T_sol/gel_ was 9.1 °C, and the flux of ciclopirox olamine from this gel matrix was calculated to be 1.44 mg/cm^2^. This emulgel of optimal composition could be used to treat fungal skin diseases.

## 1. Introduction

Pharmaceutical product development is a complex, time-consuming, and expensive process. Formulation modeling is an essential aspect of pharmaceutical product development, playing an important role in understanding and predicting the behavior of these products, ensuring their safety, efficacy and quality [1]. The integration of experimental and modeling techniques is a powerful tool in the rational design of formulation development. Response surface methodology (RSM) is a widely used technique in the design of experiments (DoE) to optimize various processes and products. Central composite design (CCD) is one of the response surface techniques that allows researchers to explore a wide range of factor combinations with few experimental runs, reducing time and resource requirements. CCD is particularly efficient for optimizing processes and products when there are multiple variables or factors to consider and allows for the identification of optimal factor settings that result in the desired response [1]. This helps in understanding how different components, such as active pharmaceutical ingredients (APIs), excipients, and various process parameters, influence the characteristics of the final product [2].

Emulgels are semi-solid pharmaceutical and cosmetic formulations that combine the properties of both emulsions and gels [3]. They have better stability compared to traditional emulsions (oil-in-water or water-in-oil) or emulsion creams. The gel structure helps prevent phase separation that can occur over time in conventional emulsion systems [4]. Both hydrophilic and lipophilic substances can be incorporated into emulgels [5]. These semi-solid forms can be formulated to provide a controlled release of active ingredients. The combination of water and oil phases in emulgels can improve the penetration of active ingredients into the skin, which can increase their effectiveness [6]. These advantages provide promising potential for application in the delivery of biologically active substances to the body.

The choice of excipients is very important when modeling a product’s composition, and excipients have a significant role in ensuring its safety, efficacy, and quality [7]. Purified water (aqueous phase), mineral oil (light) (oil phase), polysorbate 80 (nonionic surfactant), and poloxamer 407 (nonionic gelling agent) were selected as excipients for emulgel production in this study. Ciclopirox olamine (CPO) was chosen as an exemplary active ingredient.

Mineral oil (light) is chemically stable and does not oxidize or turn rancid, which can occur in some vegetable oils [8]. This stability ensures a longer shelf life for products that contain mineral oil. This oil is less likely to cause skin irritation or allergic reactions, making it suitable for use in cosmetics and skincare products.

Polysorbate 80 (Tween 80) is a commonly used nonionic surfactant that is an effective emulsifier. It can stabilize emulsion systems by helping oil and water-based ingredients mix and stay mixed. Polysorbate 80 can enhance the solubility of poorly water-soluble substances. This surfactant is considered non-toxic and safe for use in pharmaceuticals, food products, and cosmetics [9].

Poloxamer 407 (Pluronic F127) is a non-toxic and non-irritating, nonionic surfactant and gelling agent that is commonly used in pharmaceuticals and cosmetics [10]. This gelling agent forms a clear, viscous liquid at low temperatures and transitions into a gel at higher temperatures [11]. Poloxamer 407 is compatible with a wide range of pH levels, which allows it to be used in various formulations without significant pH adjustments. This polymer increases the solubility of poorly water-soluble substances [12]. Poloxamer 407 is a block copolymer composed of hydrophilic polyethylene oxide (PEO) and hydrophobic polypropylene oxide (PPO) blocks [13]. This amphiphilic structure allows it to interact with both water and oil phases in an emulsion and in such a case can stabilize these systems. Typically, the concentration of poloxamer 407 in hydrogels is in the range of 20–30%.

Ciclopirox olamine is a broad-spectrum antifungal drug commonly used to treat fungal infections of the skin and nails. It inhibits the growth of fungi by interfering with the synthesis of essential components of fungal cell membranes, disrupting their structure and function, which leads to the death of the fungus [14].

The aim of the study was to model and produce experimental emulgels containing 1% ciclopirox olamine, to evaluate their physical, rheological, and mechanical properties and their ability to release ciclopirox olamine. Based on the selected criteria, the composition of the experimental emulgel containing 1% ciclopirox olamine was optimized.

## 2. Materials and Methods

### 2.1. Chemicals and Solvents

Ciclopirox olamine (>98%) was supplied by Chemical Point (Oberhaching, Germany). Trifluoroacetic acid (99% purity, TFA), mineral oil (light), polysorbate 80, and poloxamer 407 were obtained from Sigma-Aldrich (Steinheim, Germany). Acetonitrile (HPLC, gradient grade, ≥99.9% purity) was obtained from Honeywell (Seelze, Germany).

Purified water was purified using the Thermo Scientific Pacific RO 7 purification system (Niederelbert, Germany). Deionized water was purified by GenPure Pro UV-TOC from Thermo Scientific (Langenselbold, Germany) to obtain water with a resistivity of up to 18.3 MΩcm at 25 °C.

### 2.2. Development of the Experimental Emulgel Containing Ciclopirox Olamine

#### 2.2.1. Experimental Design for Emulgel Composition Modeling and Optimization

**Figure 1:**
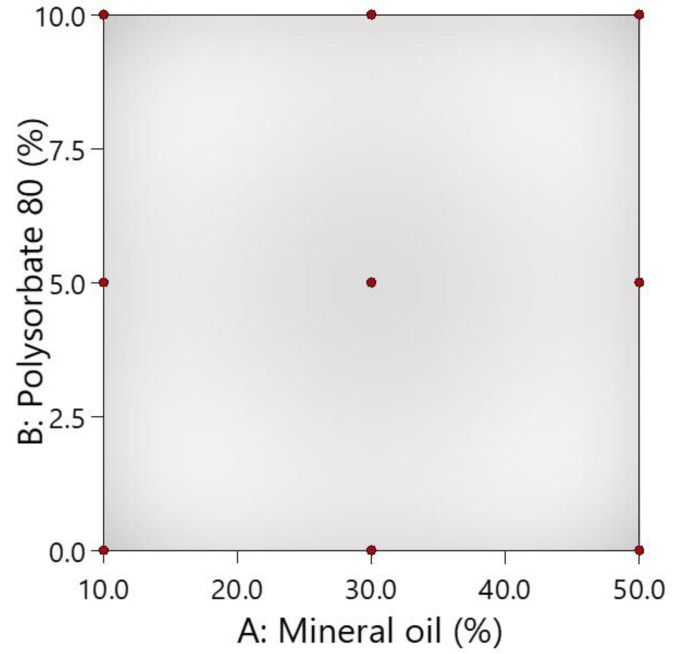
The surface area of the central composite design. The red dots indicate different compositions of emulgels.

The central composite design (CCD) model was selected as the response surface experimental design (Design-Expert 13 (Stat-Ease, Inc., Minneapolis, MN, USA)), and an image of its surface is provided in Figure 1.

Mineral oil and polysorbate 80 were selected as independent variables (factors), and their limits were as follows: 10–50% mineral oil and 0–10% polysorbate 80. Within the limits of the specified components, 9 emulgel compositions were given (Table 1).

The experimental emulgels containing 1% ciclopirox olamine were evaluated according to the following parameters (dependent variables): pH, particle size, sol–gel transition temperature (T_sol-gel_), the release flux of ciclopirox olamine, the zone size of antifungal activity, and spreadability properties (firmness and work of shear).

#### 2.2.2. Production of Poloxamer Hydrogels Containing Ciclopirox Olamine

Ciclopirox olamine, with a final concentration of 1% in the emulgels, was dissolved together with a required amount of poloxamer 407 (Table 1) in the purified water at a temperature of +4 °C. The gels were formed at room temperature.

#### 2.2.3. Production of the Experimental Emulgels

The experimental emulgels were produced using an Unguator^®^ 2100 (Gako GmbH, Munich, Germany) automatic mixer and “GEL” mode. The required amounts (Table 1) of hydrogel, mineral oil, and, if necessary, polysorbate 80 were placed in a special container. After stirring for approximately 30 min, the experimental emulgels containing 1% ciclopirox olamine were prepared. The formulations were stored at room temperature until testing.

### 2.3. Evaluation of the Physical Properties of the Experimental Emulgels

#### 2.3.1. Measurement of pH

The pH of the experimental emulgels was measured with a pH-meter (766 Callimatic (Knick, Berlin, Germany)) using an SE 104 N electrode.

#### 2.3.2. Assessment of Particle Size

The particle size of the oil phase was measured using a Mastersizer 3000 with a Hydro EV unit (Malvern Panalytical Ltd., Malvern, UK). Purified water was used as the dispersant. The sample was added so that the laser obscuration was between 9.5 and 10.5%. The pump speed was kept constant at 2400 rpm. The refractive index was 1467. One sample was tested 5 times, and the average of the measurements was calculated. The results were evaluated by analyzing the percentile (D10, D50 and D90) values.

#### 2.3.3. Assessment of the Rheological Properties

The gelation temperature was determined using an MCR102 rotary rheometer (Anton Paar BmbH, Gratz, Austria) with plate–cone geometry. A Peltier system was used to maintain the temperature. The test conditions were chosen within the viscoelastic range: angular frequency, 10 rad/s, and deformation, 0.2%. The temperature ranged from 40 to 0 °C, and the temperature change speed was 1 °C/min. The values of the storage module were used to analyze the results. The T_sol/gel_ range was considered to be the temperature interval in which the variation in G′ was the largest.

#### 2.3.4. Texture Analysis

Texture analysis was performed using a TA.XT Plus Texture Analyzer (Stable Micro Systems, Godalming, UK). The spreadability of the emulgel was evaluated according to two criteria: firmness and work of shear (distance, 10 mm; test speed, 2 mm/s; trigger force, 0.294 N (30 g)).

**Figure 2:**
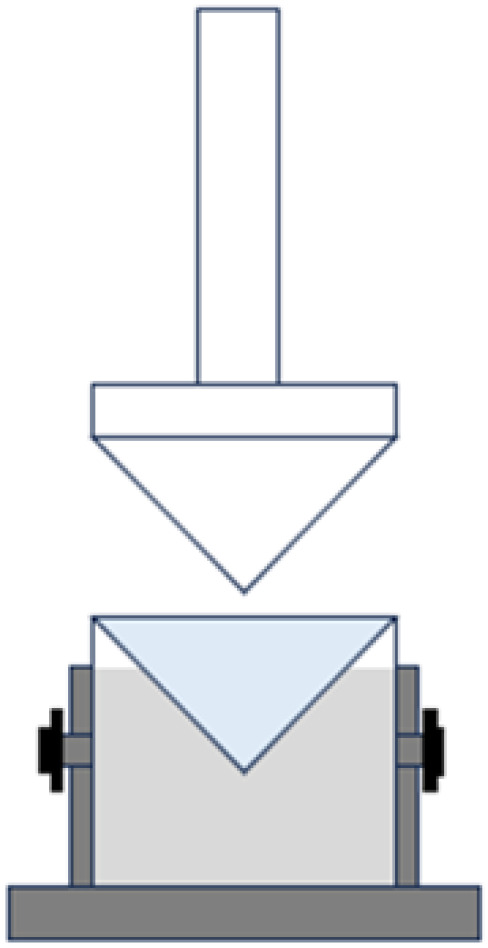
Schematic method of texture analysis.

The gel was placed in a special container (Figure 2), and the test was conducted according to the set parameters. Analyses were performed in triplicate, and the average values were calculated. The firmness meanings of back extrusion and spreadability tests were found to be varied by different probes.

### 2.4. In Vitro Release Study

An in vitro release test was performed using flow-through cells and cellulose membrane. The donor phase (1.0 ± 0.1 g) was placed in the adapter for semi-solid forms. The diffusion area was 1.33 cm^2^. Purified water (50 mL) was used as an acceptor medium (pH 5.9). The test conditions met the criteria of sink conditions. The temperature of the acceptor medium was set at 32 °C. The duration of the study was 6 h. Samples were taken every hour, and the removed volume of solution was replaced with fresh water.

All samples were filtered using a polyvinylidene difluoride filter (pore size 0.2 µm). The concentration of the active substance was determined using a liquid chromatographic system.

### 2.5. Ultra-Performance Liquid Chromatography Method for Ciclopirox Olamine Analysis

The quantitative analysis of ciclopirox olamine samples was performed using an Acquity UPLC Waters system (Waters, Milford, MA, USA) with DAD (Waters, Milford, MA, USA). The chromatographic column was C18 (Acquity UPLC^®^ BEH), 2.1 × 50 mm, and the particle size was 1.7 µm. The mobile phase consisted of 0.5% of aqueous TFA solution (60%) and acetonitrile (40%). The chromatographic conditions were as follows: an isocratic elution, a flow rate of 0.7 mL/min, a duration of analysis of 2 min, a column temperature of 25 °C, and an injection volume of 5 µL. The detection of ciclopirox olamine was at a wavelength of 303 nm, and the retention time of ciclopirox olamine was 0.941 min. The limits of the calibration graph were in the range of 4–324 µg/mL.

### 2.6. In Vitro Antifungal Test

The inhibitory activity of emulgels against zoonotic dermatophyte *Microsporum canis* was assessed by the agar well diffusion method. Five strains of *M. canis* isolated from clinical samples of cats were evaluated. The fungi suspension was prepared following the National Committee for Clinical Laboratory Standard protocol (NCCLS, 2002). The agar well diffusion test was performed using potato dextrose agar. The agar plate surface was inoculated by spreading 10 µL of the fungal inoculum over the entire agar surface. The inoculum used was prepared using the *M. canis* from a 5-day culture on Sabouraud dextrose agar; a suspension was made in a sterile saline solution. The turbidity of the suspension was adjusted with a spectrophotometer at 530 nm to obtain a final concentration to match that of a 0.5 McFarland standard (1–5 × 10^6^). A hole with a diameter of 8 mm was punched aseptically with a sterile metal pipe, and a volume of 50 µL of emulgels was introduced into the well. Each emulgel was placed on a separate plate. Agar plates were incubated at 30 °C for 5 days and observed daily. The experiment was performed in triplicate. Inhibition zone diameters were measured in millimeters. The reading of inhibition diameter zones (mm) was performed at 72 h. Zone diameters were measured to the nearest whole millimeter at a point at which there was no visible growth (100% inhibition).

### 2.7. Statistical Analysis

The results are presented as means (standard deviation). Experimental design data were evaluated by the ANOVA statistical method when *p* < 0.05.

The Mann–Whitney test was used to compare 2 independent samples. The Kruskal–Wallis test was used to compare 3 or more independent samples. The dependence of two variables was assessed using the Spearman correlation coefficient.

## 3. Results

### 3.1. Statistical Analysis ANOVA of Responses Models

The fit of the model was assessed by the *p*-value (*p* < 0.05), the difference between adjusted R^2^ and predicted R^2^ (<0.2), and the adeq precision value (>4). The values of adjusted R^2^ and predicted R^2^ should be close to 1. Adeq precision measures the signal-to-noise ratio. A ratio greater than 4 indicates that a model can be used to navigate the design space. Statistical analysis data are presented in Table 2.

Based on the results obtained for each response (dependent variable), a linear or quadratic model was suggested. The linear model of the particle size (D50), release flux, the quadratic model of pH, T_sol-gel_, the firmness of spreadability, and the work of shear were statistically significant, and these are confirmed by the *p*-value, and the fit of these models are confirmed by values of adjusted R^2^, predicted R^2^, and adeq precision (Table 2).

The model terms are considered statistically significant if the *p*-value is lower than 0.05. However, when the *p*-value is greater than 0.10, such model terms are insignificant and can be removed from the equation (not counting those required to support hierarchy), improving the model. These data are given in Table 3.

The model of each response has corresponding mathematical equations with coded or actual factors (Table 4). The equation in terms of coded factors (levels are −1, 0, +1) is used to identify the relative impact of the factors by comparing the factor coefficients. The sign + or − to the coefficient indicates a direct or inverse effect on the measured property of the system. The equation in terms of actual factors is used to make predictions about the response when the factors are expressed in their real values.

Statistical analysis ANOVA showed that factor A (mineral oil) and factor B (polysorbate 80) were statistically significant in the model (*p* = 0.0056 and *p* = 0.0256, respectively). However, mineral oil had a slightly greater influence on the pH of the emulgels according to the coefficients of the coded factors in the equation (coefficient is 0.03) than polysorbate 80 (coefficient is 0.02).

Factor A (mineral oil) and factor B (polysorbate 80) are statistically significant in the model (*p* = 0.0014 and *p* < 0.0001, respectively). However, polysorbate 80 had a greater influence on the average particle size of emulgels according to the coefficients of the coded factors in the equation (coefficient is 0.09) than mineral oil (coefficient is 0.03).

Factor B (polysorbate 80) is statistically significant in the model and had the greatest influence on the release flux of ciclopirox olamine from emulgels according to the coefficients of the coded factors in the equation (*p* = 0.0005, coefficient is 0.18). Meanwhile, factor A (mineral oil) is insignificant in this model (*p* = 0.1874, coefficient is 0.04).

Factor A (mineral oil) and factor B (polysorbate 80) are statistically significant in the model (*p* = 0.0086 and *p* < 0.0001, respectively). However, polysorbate 80 had a greater influence on the release flux of ciclopirox olamine from emulgels according to the coefficients of the coded factors in the equation (coefficient is 3.32) than mineral oil (coefficient is 1.12).

Factor B (polysorbate 80) is statistically significant in the model and had the greatest influence on the firmness of emulgels according to the coefficients of the coded factors in the equation (*p* = 0.0056, coefficient is 0.35). Meanwhile, factor A (mineral oil) is insignificant in this model (*p* = 0.5275, coefficient is 0.04).

Factor B (polysorbate 80) is statistically significant in the model and had the greatest influence on the work of shear of emulgels according to the coefficients of the coded factors in *the equation* (p = 0.0104, coefficient is 0.56). Meanwhile, factor A (mineral oil) is insignificant in this model (p = 0.6646, coefficient is 0.05).

### 3.2. The pH Evaluation of the Experimental Emulgels

**Figure 3:**
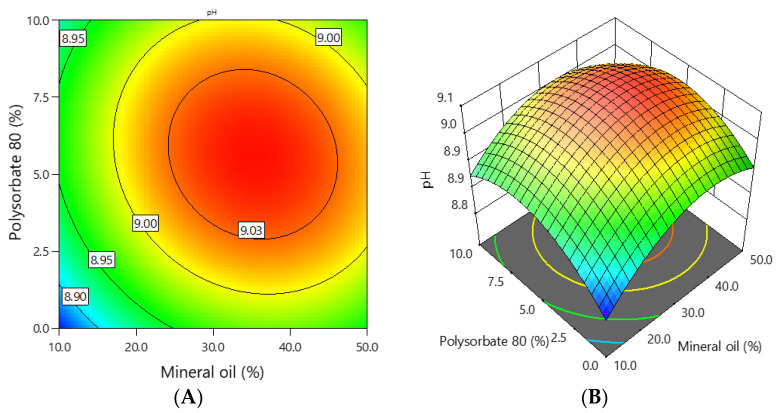
The dependence of the emulgel pH on the concentration of mineral oil and polysorbate 80: (**A**) 2D graph; (**B**) 3D graph.

The pH of the experimental emulgels containing 1% ciclopirox olamine was in the range of 8.9–9.1, and the dependence of pH on the concentration of mineral oil and polysorbate 80 is shown in Figure 3.

The graphs illustrate that the pH of the emulgels depended on the concentration of mineral oil and polysorbate 80. Formulations with higher amounts of these substances had higher pH values. Emulgels which contained about 30–40% mineral oil and 2.5–7.5% polysorbate 80 had the highest pH values.

Pharmaceutical semi-solid products should have a pH of about 4–7 to avoid having irritating effects on human skin. The pH of the produced experimental emulgels without ciclopirox olamine was in the range of 6.9–7.1. Thus, ciclopirox olamine may have influenced the alkaline pH of the emulgels. It was decided not to change the pH of the formulations at this stage of the research. Further research on these formulations will aim to adjust the pH to be suitable for use on the skin.

### 3.3. The Particle Size and Distribution Evaluation of the Experimental Emulgels

The D10, D50, and D90 percentiles indicate the size at which 10, 50, and 90 percent of the particles are equal to or less than, respectively. Table 5 shows the D10, D50, and D90 percentile values of the tested emulgels.

The D10 values ranged from 0.151 to 0.377 μm. Statistical analysis revealed that the addition of 10% polysorbate 80 statistically significantly reduced the D10 values when compared to formulations containing 5% polysorbate (*p* = 0.007) or without polysorbate 80 (*p* < 0.001). A strong negative correlation was found between polysorbate 80 content and D10 values (r = −0.822, *p* < 0.001). Meanwhile, formulations with different oil content were not statistically significantly different. The relationship between oil content and D10 values was weak (r = −0.455, *p* = 0.017).

The D50 values ranged from 0.240 to 0.512 μm. The addition of 10 % polysorbate 80 statistically significantly reduced the D50 values when compared to formulations containing 5% polysorbate (*p* = 0.038) or without it (*p* < 0.001). A very strong negative correlation was found between polysorbate 80 content and D50 values (r = −0.910, *p* < 0.001). Formulations with different oil content were not statistically significantly different. The relationship between oil content and D50 values was weak (r = −0.356, *p* = 0.069).

D90 was in the range from 0.413 to 1.002 μm. The addition of 5 or 10 % polysorbate statistically significantly changed the D90 values compared to formulations without polysorbate (*p* = 0.048 and *p* < 0.001, respectively). A very strong negative correlation was found between polysorbate 80 content and D10 values (r = −0.944, *p* < 0.001). Meanwhile, formulations with different oil content were not statistically significantly different. The relationship between oil content and D10 values was very weak (r = −0.292, *p* = 0.014).

**Figure 4:**
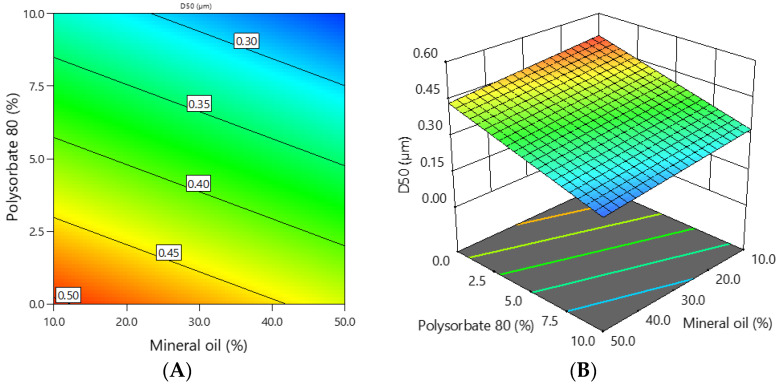
The dependence of the average particle size of the emulgel on the concentration of mineral oil and polysorbate 80: (**A**) 2D graph; (**B**) 3D graph.

Figure 4 depicts the relationship between the average particle size and the concentrations of mineral oil and polysorbate 80.

The average particle size of the emulgels depended on the concentration of mineral oil and polysorbate 80. The coefficients of the mathematical equation of coded factors (Table 4) show that polysorbate 80 had a stronger effect on the average particle size of the emulgels than mineral oil.

### 3.4. The Evaluation of the Rheological Properties of the Experimental Emulgels

**Figure 5:**
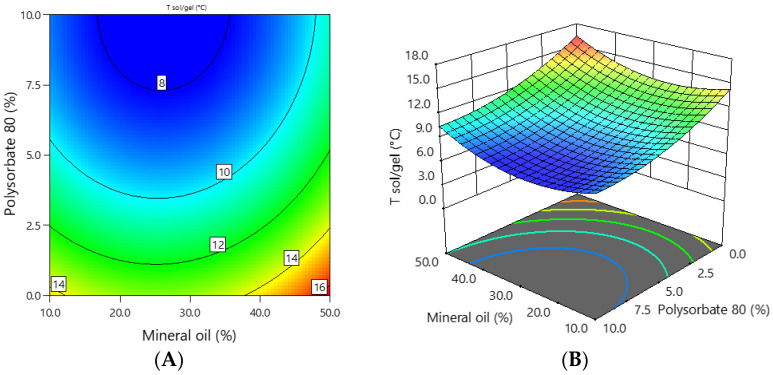
The dependence of the sol–gel transition temperature of the emulgel on the concentration of mineral oil and polysorbate 80: (**A**) 2D graph; (**B**) 3D graph.

The sol–gel transition temperature (T_sol-gel_) of the experimental emulgels containing 1% ciclopirox olamine was in the range of 8.0–16.0 °C, and the dependence of the sol–gel transition temperature on the concentration of mineral oil and polysorbate 80 is shown in Figure 5.

The lowest T_sol-gel_ temperature was found for formulation E-4 containing 30% oil and 10% polysorbate, and the highest was found for emulgel E-8 containing 50% oil and no polysorbate 80. This was confirmed by statistical analysis, which showed a statistically significant (*p* < 0.01) inverse strong (r = −0.820) correlation between the concentration of polysorbate 80 and T_sol-gel_ of the emulgels. The amount of oil did not affect the T_sol-gel_ temperature (r = 0.212, *p* = 0.585). The coefficients of the mathematical equation of coded factors (Table 4) show that polysorbate 80 had a stronger effect on the sol–gel transition temperature of the emulgels than mineral oil.

### 3.5. The Evaluation of the Texture Analysis of the Experimental Emulgels

**Figure 6:**
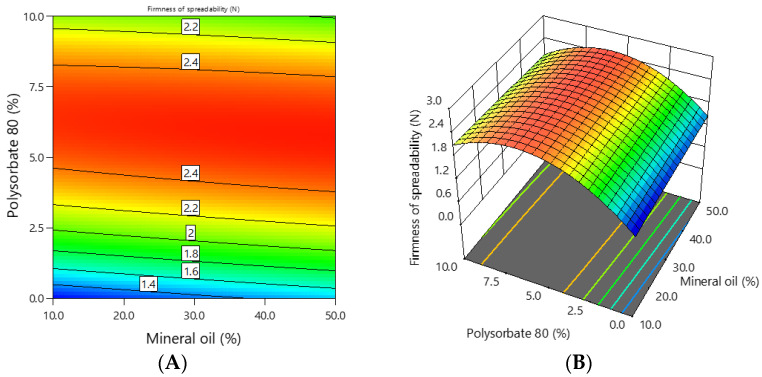
The dependence of the firmness of the emulgel on the concentration of mineral oil and polysorbate 80: (**A**) 2D graph; (**B**) 3D graph.

The firmness of the experimental emulgels containing 1% ciclopirox olamine was in the range of 1.2–2.6 N during spreadability, and the dependence of the firmness on the concentration of mineral oil and polysorbate 80 is shown in Figure 6.

It can be seen from the graphs that the firmness of the emulgels depended on the concentration of polysorbate 80. The higher the concentration of polysorbate 80 in the emulgel, the higher the firmness. The coefficients of the mathematical equation of coded factors (Table 4) show that polysorbate 80 had a stronger effect on the firmness of the emulgels than mineral oil.

**Figure 7:**
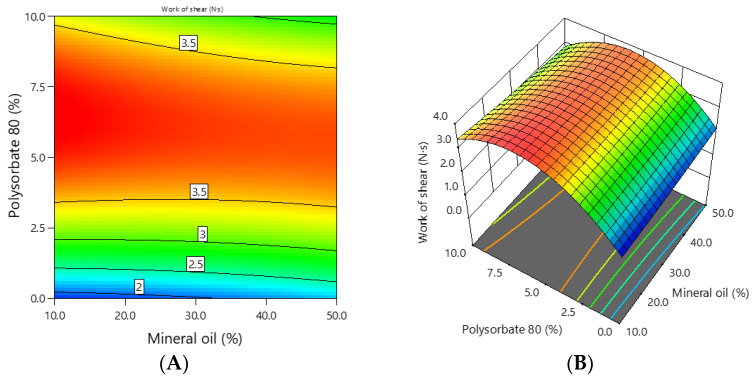
The dependence of the shear work of the emulgel on the concentration of mineral oil and polysorbate 80: (**A**) 2D graph; (**B**) 3D graph.

The shear work of the experimental emulgels containing 1% ciclopirox olamine was in the range of 1.8–4.0 N·s during spreadability, and the dependence of the shear work on the concentration of mineral oil and polysorbate 80 is shown in Figure 7.

It can be seen from the graphs that the shear work of the emulgels depended on the concentration of polysorbate 80. This was confirmed by the previously mentioned statistics (Table 2). The higher the concentration of polysorbate 80 in the emulgel, the higher the shear work. The coefficients of the mathematical equation of coded factors (Table 4) showed that polysorbate 80 had a stronger effect on the shear work of the emulgels than mineral oil.

### 3.6. The Evaluation of Ciclopirox Olamine Release from the Experimental Emulgels

**Figure 8:**
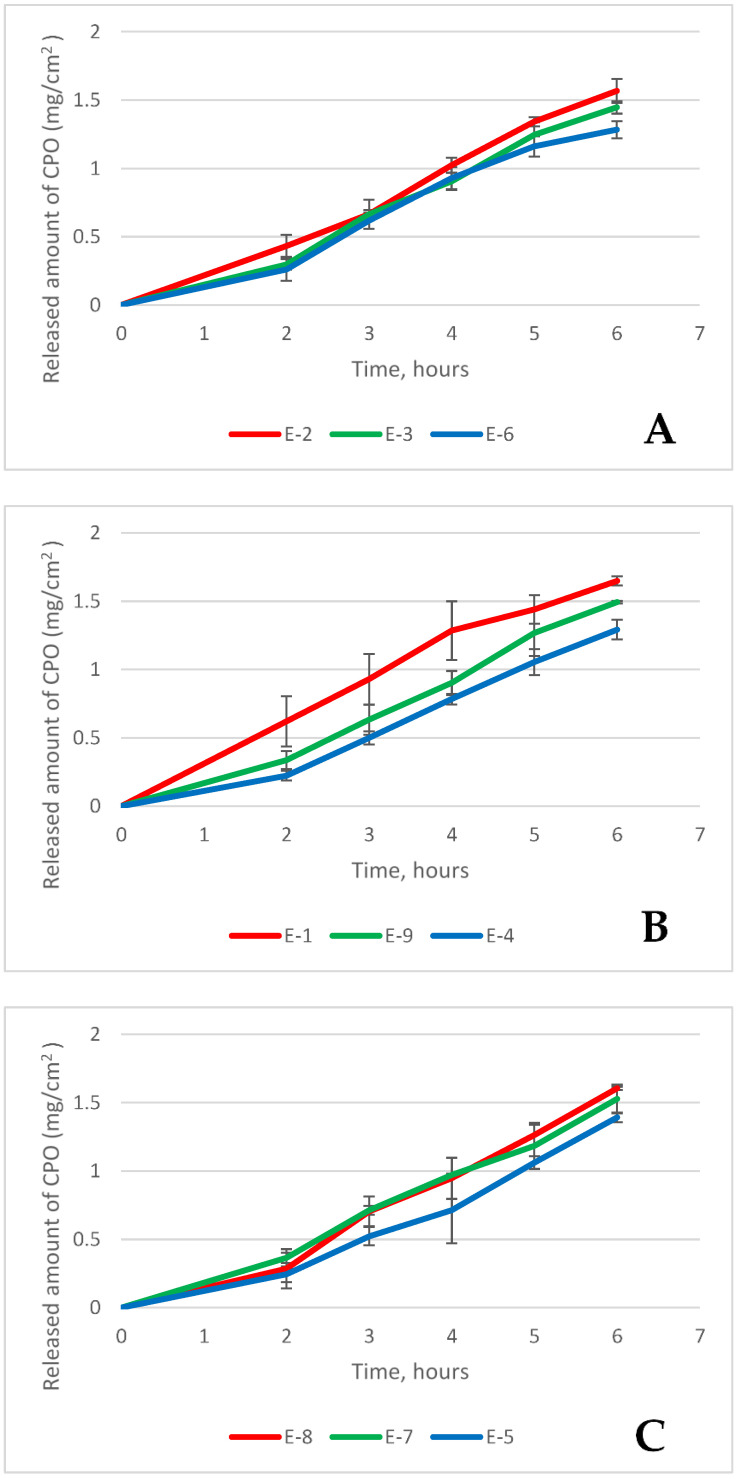
The profiles of ciclopirox olamine release from emulgels: (**A**) emulgels with 10% oil, (**B**) emulgels with 30% oil, (**C**) emulgels with 50% oil.

Release studies are important in the study of pharmaceutical formulations. The release of the active ingredient can ensure the proper functioning of the drug. The release kinetics plots are shown in Figure 8.

Figure 8A shows release kinetic plots from formulations containing 10% oil but varying amounts of polysorbate 80. The addition of polysorbate 80 changed the release flux of ciclopirox olamine after 6 h: a statistically significant difference was found between the formulation with 10% polysorbate 80 and the formulation without polysorbate 80 (*p* = 0.022). A very strong negative correlation was found between polysorbate content and the release flux of ciclopirox olamine (r = −0.949, *p* < 0.001).

Figure 8B shows release kinetic plots from formulations containing 30% oil. It was found that 10% polysorbate statistically significantly altered the released amount of ciclopirox olamine after 6 h compared to the emulgel without polysorbate (*p* = 0.022). A statistically significant and highly strong correlation was observed between the amount of polysorbate and the release flux (r = −0.949, *p* < 0.001).

Figure 8C shows release kinetic plots from formulations containing 50% oil. No statistically significant differences were found between emulgels with different amounts of polysorbate 80. A moderate statistically significant correlation between the amount of polysorbate and the released amount was determined (r = −0.685, *p* = 0.042). Polysorbate 80 had less influence on the released amount of ciclopirox olamine when the oil content was 50%.

**Figure 9:**
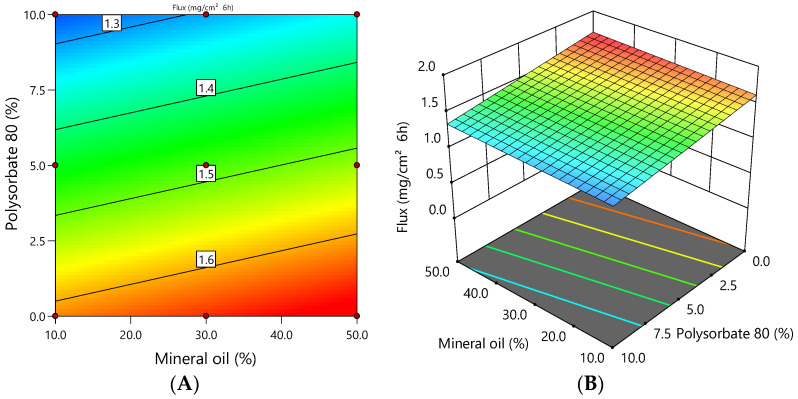
The dependence of the release flux of ciclopirox olamine from the emulgel on the concentration of mineral oil and polysorbate 80: (**A**) 2D graph; (**B**) 3D graph.

It can be seen from Figure 9 that the release flux of ciclopirox olamine from the emulgels depended on the concentration of polysorbate 80. The lower the concentration of polysorbate 80 in the emulgel, the higher the release flux of ciclopirox olamine. The coefficients of the mathematical equation of coded factors (Table 4) showed that polysorbate 80 had a stronger effect on the release flux of ciclopirox olamine from the emulgels than mineral oil. This was confirmed by statistical analysis, which showed a statistically significant (*p* < 0.01) inverse very strong (r = −0.949) correlation between the concentration of polysorbate 80 and the release flux of ciclopirox olamine from the emulgels.

### 3.7. The Evaluation of Antifungal Activity of the Experimental Emulgels

The average inhibition zone diameter of all tested emulgels was 47.82 (2.36) mm, with 50.8 (1.78) mm for the E-8 emulgel and 43.4 (2.51) mm for the E-7 emulgel. Control emulgels without the pharmaceutical substance showed no inhibition. Unfortunately, it was not possible to determine the difference in the rate of penetration of emulgels in agar. We assume that the components of the emulgel distribute faster in the agar than the observed growth of the fungus.

## 4. Discussions

The surface response central composition design is one of the experimental design models that allows for a purposeful search for the optimal product composition according to selected criteria [1,15]. This technique reduces the number of experiments when studying the relationship between several variables and saves experimental time and human and natural resources. In order to model emulgels, two variables were selected: oil content and polysorbate 80 content.

The particle size of the oil phase is an important parameter that can affect the stability of the emulsion system and the penetration of the active substance into human skin. Large particles tend to bond, and this leads to phase separation. An emulsifier forms a layer at the interface, so it is important that the amount of emulsifier in an emulgel is sufficient [16].

This study found that 10 percent polysorbate content resulted in a statistically significant reduction in particle size. This could be attributed to more stable droplet formation. These findings align with other scientists’ research results. Patricia Tello et al. [5] found that the amount of phytocyanin as an emulsifier affected the particle size of emulsifiers, with an increasing emulsifier concentration decreasing particle size.

Rheological properties affect the release of the active ingredient from formulations and absorption at the site of action; thus, determining the rheological properties is very important [17]. The sol–gel transition temperature is another important parameter indicating the equilibrium between liquid and gel systems at a given temperature. The lower the temperature, the more stable the semisolid system is over a wider temperature range. This is especially relevant when using poloxamer 407 as a gelling agent as semi-solid systems with this component are sensitive to temperature changes. A statistically significant negative correlation was found between polysorbate 80 concentration and T_sol-gel_. Oil content shows no statistically significant influence on rheological properties.

A material’s resistance to deformation is referred to as its firmness [18]. It describes how easily an emulgel spreads or flows when applied on the skin. Better spreadability is indicated by a lower firmness value [19].

When polysorbate 80 content was between 5 and 7.5%, the highest firmness of emulgels was determined. Poloxamer 407 concentration may have an impact on the solidity of emulgels when using less than 5 and more than 7.5% polysorbate 80. The firmness of the emulgel was at its highest when using 12.5%, and 22.5% poloxamer 407 and 50% and 10% mineral oil, respectively; however, the firmness of emulgels was at a medium value when using 17.5% poloxamer. In the micelle shell, poloxamer molecules are interrupted by polysorbate molecules. It is widely known that the synergistic impact of amphiphilic substances and polysorbate produces stronger micelles than poloxamers alone [20,21].

In another study, the firmness of an emulgel used for psoriasis was 4 N (three times less than hydrogel; 3% carbopol 980 as gelificator and 4% polysorbate 80 were used) [6]. A nanoemulsion-based emulgel with polysorbate 80, essential oils, and carbopol 940 in its composition showed a similar firmness (1.1 N) to the emulgel used in this investigation with 1% ciclopirox olamine [22].

The amount of energy needed to deform an emulgel is measured by the work of shear. It measures the effort required to determine the emulgel’s internal structure and promote spread. A higher work of shear suggests that more energy is needed to achieve proper spreadability [23,24].

Scientists can analyze the structural stability and flow behavior of emulgels by examining both parameters. In general, lower values for stiffness and work of shear imply that the emulgel formulation has good spreadability and is likely to be applied and spread on the skin without difficulty [18]. This knowledge is essential for improving emulgel formulations for use in a variety of products, including medications and cosmetics [24].

The physical characteristics of bigels were significantly impacted by the rising lipophilic phase content (oil and emulsifier) [25,26]. Higher oil and polysorbate 80 concentrations were also discovered to affect firmness and shear work in this study. Despite the fact that Figure 8 and Figure 9 show that the physical characteristics of the texture decreased when the emulsifier content was above 7.5% (less poloxamer, a gelling agent, was used in the composition, which also influenced the bigel’s structure).

The flux of CPO from emulgels demonstrates how efficiently the semi-solid system can release the active substance [4]. The kinetics graphs of the release studies show that the release of ciclopirox olamine from the tested emulgels occurred according to first-order kinetics (R^2^ was from 0.9615 to 0.9908). A statistically significant (*p* < 0.01), direct strong (r = 0.817) correlation was found between the release flux of ciclopirox olamine from the emulgels and the average particle size (D50). A statistically significant (*p* < 0.01), direct strong (r = 0.812) correlation was found between the release flux of ciclopirox olamine from the emulgels and T_sol-gel_ of the emulgels. This indicates that the release of ciclopirox olamine from emulgels increases with the increasing oil particle size and sol–gel transition temperature (T_sol-gel_) of these semi-solid forms.

*Microsporum canis* is a common dermatophyte found in animals and humans. Improper hygiene, closed footwear, diabetes mellitus, age, genetics, and immunocompromised status can increase the risk of infection [27]. *M. canis* can cause various fungal infections: tinea capitis, tinea corporis, tinea pedis, and onychomycosis [28]. Ciclopirox olamine is effective against a variety of fungi, including *M. canis*. Ciclopirox olamine is used in various forms: creams, lotions, topical suspensions, gels, and nail polish [29]. Ciclopirox olamine is effective against a variety of fungi. The efficacy of ciclopirox olamine against fungi found in this study replicates previous results obtained by this group of researchers [30,31,32] and agrees with the other scientists’ results.

In this study, the maximum CPO flux from the investigated emulgels, the minimum sol–gel transition temperature (T_sol/gel_), and the minimum particle size of the oil phase were chosen as the selection criteria for the optimal composition of emulgels. According to these criteria, during optimization, an emulgel of optimal composition was generated, containing 38.27% mineral oil, 6.56% polysorbate 80, and 55.17% hydrogel containing 1% CPO. The desirability value of the optimization process was 0.646. This shows that the obtained optimal emulgel composition met the selected criteria conditions by 64.6%. The oil phase particle size (D50) of this emulgel should be 0.337 µm, the system T_sol/gel_ should be 9.1 °C, and the flux of CPO from this gel matrix should be 1.44 mg/cm^2^.

## 5. Conclusions

The design of the surface response center composition chosen during the experimental planning allowed for the modeling of experimental emulgels with a 1% ciclopirox olamine formulation and reduced the number of experiments by generating nine formulations. The surfactant (polysorbate 80) had the greatest influence on the physical, rheological, and mechanical properties of emulgels and the release of ciclopirox olamine from these systems. Mineral oil had no or weak influence on the previously mentioned properties. The modeled semisolid systems did not reduce the antifungal activity of ciclopirox against *M. canis*. The selected criteria allowed for the purposeful selection of the optimal composition of experimental emulgels, which could be used for the treatment of fungal skin diseases. The results of the studies have shown how important it is to select the right materials and their concentrations to produce a quality product.

## Figures and Tables

**Table 1:** The percentage compositions of the experimental emulgel components.

Emulgels No.	Independent Variables		Hydrogel		
	Factor A	Factor B			
	Mineral Oil (%)	Polysorbate 80 (%)	Poloxamer 407 (%)	Purified Water (%)	Ciclopirox Olamine (%)
E-1	30.0	–	17.5	51.5	1.0
E-2	10.0	–	22.5	66.5	1.0
E-3	10.0	5.0	22.5	61.5	1.0
E-4	30.0	10.0	17.5	41.5	1.0
E-5	50.0	10.0	12.5	26.5	1.0
E-6	10.0	10.0	22.5	56.5	1.0
E-7	50.0	5.0	12.5	31.5	1.0
E-8	50.0	–	12.5	36.5	1.0
E-9	30.0	5.0	17.5	46.5	1.0

**Table 2:** Statistical analysis ANOVA data of the fit of the model.

Response	Model	*p*-Value	Adjusted R^2^	Predicted R^2^	Adeq Precision
pH	quadratic	0.0065	0.9584	0.8385	20.2
Particle size (D50)	linear	<0.0001	0.9694	0.9472	29.1
T_sol-gel_	quadratic	0.0154	0.9255	0.7188	13.6
Release flux	linear	0.0015	0.8482	0.7165	11.5
Firmness of spreadability	quadratic	0.0103	0.9433	0.7449	13.2
Work of shear	quadratic	0.0189	0.9147	0.6104	10.3

**Table 3:** The significant data of the model terms.

Response	A (Mineral Oil)	B (Polysorbate 80)	AB	A^2^	B^2^
pH	*p* = 0.0056	*p* = 0.0256	*p* = 0.1045	*p* = 0.0047	*p* = 0.0047
Particle size (D50)	*p* = 0.0014	*p* < 0.0001	–	–	–
T_sol-gel_	*p* = 0.0659	*p* = 0.0031	*p* = 0.7730	*p* = 0.0338	*p* = 0.0976
Release flux	*p* = 0.1874	*p* = 0.0005	–	–	–
Firmness of spreadability	*p* = 0.5275	*p* = 0.0056	*p* = 0.2035	*p* = 0.9281	*p* = 0.0028
Work of shear	*p* = 0.6646	*p* = 0.0104	*p* = 0.1765	*p* = 0.8000	*p* = 0.0052

**Table 4:** The mathematical equations of significant models.

Response	The Equations	
	Coded Factors ^1^	Actual Factors ^1^
pH	Y = 9.04 + 0.03A + 0.02B − 0.01AB − 0.06A^2^ − 0.06B^2^	Y = 8.7681 + 0.0110X_1_ + 0.0308X_2_ − 0.00013X_1_X_2_ − 0.00015X_1_^2^ − 0.0023X_2_^2^
Particle size (D50)	Y = 0.38 − 0.03A − 0.09B	Y = 0.5209 − 0.0017X_1_ − 0.0181X_2_
T_sol-gel_	Y = 9.11 + 0.92A − 2.83B − 0.13AB + 2.08A^2^ + 1.33B^2^	Y = 16.4028 − 0.2604X_1_ − 1.0625X_2_ − 0.0013X_1_X_2_ + 0.0052X_1_^2^ + 0.0533X_2_^2^
Release flux	Y = 1.48 + 0.04A − 0.18B	Y = 1.5979 + 0.0020X_1_ − 0.0352X_2_
Firmness of spreadability	Y = 2.48 + 0.04A + 0.35B − 0.10AB − 0.01A^2^ − 0.78B^2^	Y = 1.1347 + 0.0079X_1_ + 0.4109X_2_ − 0.0010X_1_X_2_ − 0.00002X_1_^2^ − 0.0311X_2_^2^
Work of shear	Y = 3.78 − 0.05A + 0.56B − 0.21AB + 0.05A^2^ − 1.24B^2^	Y = 1.8356 + 0.0012X_1_ + 0.6723X_2_ − 0.0021X_1_X_2_ + 0.00012X_1_^2^ − 0.0497X_2_^2^

^1^ A and X_1_ are mineral oil, B and X_2_ are polysorbate 80, Y is the appropriate response.

**Table 5:** Percentile (D10, D50, D90) values. The results are presented as means (standard deviations).

Formulation	D10	D50	D90
E-1	0.309 (0.011)	0.461 (0.009)	0.900 (0.010)
E-2	0.377 (0.043)	0.512 (0.019)	1.002 (0.050)
E-3	0.338 (0.043)	0.422 (0.032)	0.737 (0.029)
E-4	0.192 (0.016)	0.295 (0.015)	0.499 (0.015)
E-5	0.151 (0.010)	0.240 (0.0.16)	0.413 (0.008)
E-6	0.221 (0.010)	0.336 (0.015)	0.557 (0.011)
E-7	0.252 (0.015)	0.374 (0.026)	0.659 (0.027)
E-8	0.282 (0.013)	0.434 (0.028)	0.884 (0.014)
E-9	0.252 (0.020)	0.381 (0.028)	0.664 (0.021)

## Data Availability

Data are available in a publicly accessible repository.
